# Group-based patient education delivered by nurses to meet a clinical standard for glaucoma information provision: the G-TRAIN feasibility study

**DOI:** 10.1186/s40814-018-0313-5

**Published:** 2018-07-03

**Authors:** H. Waterman, S. Bull, M. Shaw, C. Richardson

**Affiliations:** 1 School of Healthcare Sciences, University of Cardiff, Eastgate House, 35-43 Newport Road, Cardiff, CF24 0AB UK; 2 McCann Health, London, UK; 3 School of Nursing, Midwifery and Social Work, University of Manchester, Manchester, UK; 4 Manchester Academic Health Sciences Centre, Manchester, UK

**Keywords:** Glaucoma, Group-based education, Adherence, Nurse, Patient compliance, Clinical guidelines, Clinical standards

## Abstract

**Background:**

Globally, glaucoma is the leading cause of irreversible blindness. However, many patients with glaucoma do not understand their disease which reportedly impacts on their ability to manage their condition successfully. The aim of this feasibility study was to undertake research to inform a future randomised controlled trial of the effectiveness of group-based education for patients to improve adherence to glaucoma eyedrops.

**Methods:**

Key objectives were to understand current provision of information during routine nurse-led glaucoma consultations, to investigate if it is possible to deliver patient information in line with a clinical standard by training nurses to deliver group-based education in multiple hospital sites and to explore the acceptability of group-based education to nurses and patients compared with usual information provision in consultations. This study employed quantitative and qualitative research methods situated in a sequential design across three hospitals in England and Wales. Current provision of information given to 112 patients with glaucoma across the three hospitals was observed and compared to that recommended in a clinical standard. Then, six nurses were trained to deliver group-based education. Following which, the level of information was assessed again in line with the clinical standard as the group-based education programme was delivered in the three hospitals to 16 patients in total. All nurses and six patients were interviewed to explore experiences of the group-based education sessions.

**Results:**

The main area of information provided during routine nurse-led consultations concerned the management of glaucoma and that least covered was about prognosis of the disease and information about support services. Nurses were trained to implement group-based education for patients. Information was provided more often by the nurses about all the items of the clinical standard when delivering group based education. However, patients’ motivation to attend were negatively impacted mainly by delays in delivering the education for the third phase and because the majority were established patients. Nurses and patients who participated found it useful and comprehensive.

**Conclusion:**

This feasibility study demonstrates that the proposed intervention for the randomised controlled trial, the group-based education, goes beyond current information provision, is in line with that articulated by a clinical standard and is implementable across several sites. This bodes well for a future randomised trial, but the following needs to be taken into account to ensure success: independent implementation of the research, training and delivery at each site, timely provision of the patient education, inclusion of new patients, and consistently delivered nurse training.

**Trial registration:**

International Standard Randomised Controlled Trial number, ISRCTN91188805

## Background

Our previous research focusses on defining the parameters needed to design a randomised controlled trial of a group-based education intervention to improve adherence to glaucoma eyedrops [1, 2]. This included work to identify the willingness of patients to be recruited and consent to participate in the education and clinicians’ willingness to approach potential participants. This is a follow-up feasibility study which aims to gather further data on the scope of the problem of information provision to patients with glaucoma and the practicality of implementing the education successfully in sites other than where it originated.

Poor awareness and understanding of glaucoma is a global challenge [3, 4]. Glaucoma is defined as a group of diseases usually associated with a rise in eye pressure and optic nerve damage. In a minority of cases, it can lead to severe vision loss. It is the leading cause of permanent loss of sight worldwide [5]. In the United Kingdom (UK), the National Institute for Clinical Excellence (NICE) published a guideline on the diagnosis and treatment of chronic open-angle glaucoma and ocular hypertension, that states people should be offered ‘the opportunity to discuss their diagnosis, prognosis and treatment, and [be] provide[d] … with relevant information in an accessible format at initial and subsequent visits including for example advice that loss of vision is permanent’ p12, [6]. This guideline has been reiterated in the NICE quality statement 11: information for glaucoma in adults [7]. The Royal College of Ophthalmologists of the UK (2016) commissioning guide for glaucoma recommend that services should be commissioned which adhere to this quality statement to provide information [8]. No other national or international quality standards for information provision could be located. The clinical standard was written in English and appeared to be applicable to all glaucoma patients regardless of which country they live in although this would need to be tested through further research. Even though this clinical standard is in existence, there are concerns in the UK and worldwide that the amount and depth of information provided to patients with glaucoma are variable and often insufficient across clinics and individual clinicians. For example, in one eye hospital across different clinics, patients were advised of the timing of eyedrops by doctors in 29/54 (64%) of consultations and 12/13 (92%) by optometrists [9]. There is no research which has considered whether nurses conform to the quality standards of information provision with regard to UK NICE glaucoma clinical guideline, hereafter referred to as the ‘clinical standard’.

The most common way to deliver information to patients is through educational interventions. Studies have shown that educational interventions may lead to better rates of adherence among patients with glaucoma, better knowledge, better perceived control over their condition and ‘stronger beliefs about the need for eyedrops’ [10]. Over one million patients attend glaucoma out-patient departments a year in the UK [6] so group-based education could be a feasible and economical method of delivery of information to patients. Our previous research has shown that group-based education is an acceptable mode for patients with glaucoma. For example, one study reported that out of 59 patients approached to participate in group-based education, about half 26 agreed to participate and of those 21 actually attended [1]. Group-based education does not preclude other formats such as one to one provision of information or web-based education but should be viewed as complimentary to these methods of delivery.

Through prior research, we determined the learning outcomes, content and mode of delivery of a group-based education programme for patients with glaucoma called the ‘Get a grip on your glaucoma’ course [11]. Patients, nurses, optometrists and doctors were directly involved in the research and creation of this programme [11]. The content of the programme included what is glaucoma, type and side effects of glaucoma, how glaucoma effects sight, eyedrop instillation technique, discussion of adherence, taking control of their condition, driving and glaucoma, and life style and glaucoma [11]. Our previous pilot study has shown that the group-based education programme appears to increase patients’ understanding of the condition, empowered patients to care for themselves regarding their glaucoma and helped patients stay adherent to medication [1]. However, there is no evaluation of the implementation of group-based education for patients with glaucoma in how it could assist nurses in the delivery of information provision as per the clinical standard and whether it could be rolled out successfully at multiple hospital sites. These are important considerations if the programme is to be tested for its effect on adherence to glaucoma eyedrops via a multi-centre randomised controlled trial.

## Methods

### Aims and objectives

The overarching aim was to determine the feasibility of delivering a group-based education intervention to patients with glaucoma in advance of a randomised controlled trial. The objectives of the research are:

1. To understand current provision of information during routine nurse-led glaucoma consultations
2. To investigate if it is possible to deliver patient information in line with a clinical standard by training nurses to deliver group-based education in multiple sites
3. To explore the acceptability of a group-based education programme to nurses and patients compared with usual information provision in consultations

### Ethics

The ethics reference number was 12/NW/0259. Informed written consent was taken from all participants.

The study was organised into three phases.

#### Phase one—observation of standard practice

##### Participants

For the first phase of the research, current clinical practice was observed at three NHS trusts/health board from England and Wales to see if patients received information as per the clinical standard for glaucoma information provision [7]. These trusts/health boards were selected because they represented both rural and urban eye hospitals with range of patient demographics. Patients with chronic open-angle glaucoma (COAG), ocular hypertension (OHT) or normal tension glaucoma, prescribed ocular hypotensive eyedrops, aged over 18 years and able to understand English to be able to give consent or have an interpreter were recruited to the study. Participants were excluded if they had a diagnosis of angle closure glaucoma or diabetic retinopathy or were not prescribed ocular medication.

##### Sample size

The intended sample size was to observe all nurses (*n* = 9) from the three hospitals who delivered nurse-led glaucoma consultations and 50 patients with glaucoma per institution. These samples were considered to be sufficient to provide a general picture of information provision during routine nurse-led glaucoma consultations.

##### Method

Patients were approached by clinic nurses and if interested in taking part in the study were given a participant information sheet to read. Informed written consent was obtained in all cases. In order to describe the sample, baseline data were collected at the clinic: age, sex, diagnosis, visual field loss and employment. Then, their medical consultation with the nurse was observed using the Provision of Vision Information Measurement Tool (VIM, Appendix) based on the key criteria in the clinical standard [7]. All the nurses whose medical consultations were observed also gave informed consent. In order to ensure consistency of observations, two researchers doubled up in observations across five early consultations. In all five consultations, the scoring was consistent except for two occasions where one did not tick a box and another did. This was resolved by discussion, and thereafter, the same principles were followed.

##### Data analysis

VIM scores were reported descriptively using total scores and percentages.

#### Phase 2—training of nurses to deliver group-based education to patients with glaucoma

##### Participants and sample size

Two nurses from each trust who had been involved in phase 1 of the study and who (i) worked with glaucoma patients and (ii) had an interest in developing their patient education skills were approached by their managers to take part in phase 2. Two nurses from each hospital were invited to minimise service disruption and to minimise risk to the study if one should drop out.

##### Method

Following informed written consent, a 2-day interactive workshop was held in which the structure and content of the ‘Get a grip on your glaucoma’ course was reviewed, updated and revised by the nurses and research team, and then, a training package for nurses was generated and implemented. It comprised of power point slides, role play and interactive sessions. The group-based education programme for patients was revised to include advice to patients about the prescription of off-patent prostaglandins so to help them understand how to deal with differing packaging and storage instructions for what is the same eyedrop.

#### Phase 3—delivery and evaluation of group-based education

##### Participants

Only those patients in phase 1 were invited to participate in phase 3, but they had to have agreed to be contacted again about attending the group-based education. Those nurses who had participated in phase 2 were invited to take part in phase 3. All participants gave informed consent.

##### Method

The group-based education sessions were observed using the same VIM-structured observation tool used during phase 1 of the study. Patients and all the nurses were also invited to be interviewed about their experiences and perspectives of the education after the programme had completed.

##### Sample size

The focus here is on whether all the nurses could deliver the group-based education; thus, we needed sufficient patients to attend a programme (see below for detail) in each hospital to be able to demonstrate that we had successfully implemented it at the three hospital sites.

##### Education programme for patients with glaucoma

The ‘Get a grip on your glaucoma’ group-based education programme was then implemented by nurses with glaucoma patients [1, 11]. The course is an education and training programme consisting of two 2-h sessions, 1 week apart. The content, length, number and frequency of sessions of the programme were guided by feedback from patients [11]. Various types of media are used during the sessions including power point presentation, handouts in large font, video, models and skill-based learning. Briefly, the first session begins with introductions and an opportunity for patients to talk about how they came to be referred to the hospital. This helps to relax patients and to make them feel comfortable talking among one another. They are asked to identify questions which they would like answering during the sessions. These are kept and reviewed as the session progresses. This also gives the session moderator insight into patients’ prior learning on glaucoma. Next, different diagnoses are explained and patients’ understanding of their own diagnosis (if they have one) are checked (this is done confidentially). Using large plastic eye models, the anatomy, physiology and pathology of glaucoma is explained in language understood. They are then shown the different techniques available for instilling eyedrops, and the eyedrop aids which can help those with physical disabilities. Then, each person’s personal eyedrop instillation technique is checked; meanwhile, the rest of patients take a break. Following on, information on the types and purposes of eyedrops is provided. Time is taken to check patients know eyedrops are for life, which eyedrops they have been prescribed, how to get repeat prescriptions and that they know how to find out how to store them. Having now a better understanding of the condition and its treatment, patients are next asked to identify barriers to drop instillation every day and on time using an adaptation of Vandenbroeck’s tool [12]. Patients also share among the group what they personally do to remain adherent. Then, they are invited to plan how they might change their daily routine to help them adhere more. This could comprise of setting reminders, thinking ahead for repeat prescriptions, purchasing cool bags for holidays.

The following week, knowledge and skills from the previous week are reviewed and any outstanding questions answered. Next, an explanation of the DVLA regulations and glaucoma is covered. Although, this may not be directly related to adherence, it is included because patients have reported that they worry about it. Then, since patients have a better understanding of their condition, patients are helped to think about what questions they would like to ask their doctor about glaucoma. NHS guidance is used to do this. Then, their barriers to adherence are reviewed and an action plans revised where appropriate to maintain adherence. Finally, patients are asked to complete an evaluation of the programme.

##### Data analysis

Descriptive statistics (total scores and percentages) were employed again to analyse the VIM data. Framework analysis was used to analyse the anonymised transcribed qualitative interview data from patients and nurses [13, 14]. Framework analysis was selected as it offers a systematic and transparent method for the analysis of qualitative data where the sample and areas for exploration are pre-determined. It comprises of five stages: (1) familiarisation with the data, (2) identification of themes or issues (3) indexing of these on the interview transcripts, (4) charting or grouping of themes and (5) mapping and interpretation of themes [13, 14].

## Results

The results are presented sequentially through phase 1 to phase 3.

### Phase 1—current provision of information

**Fig. 1:**
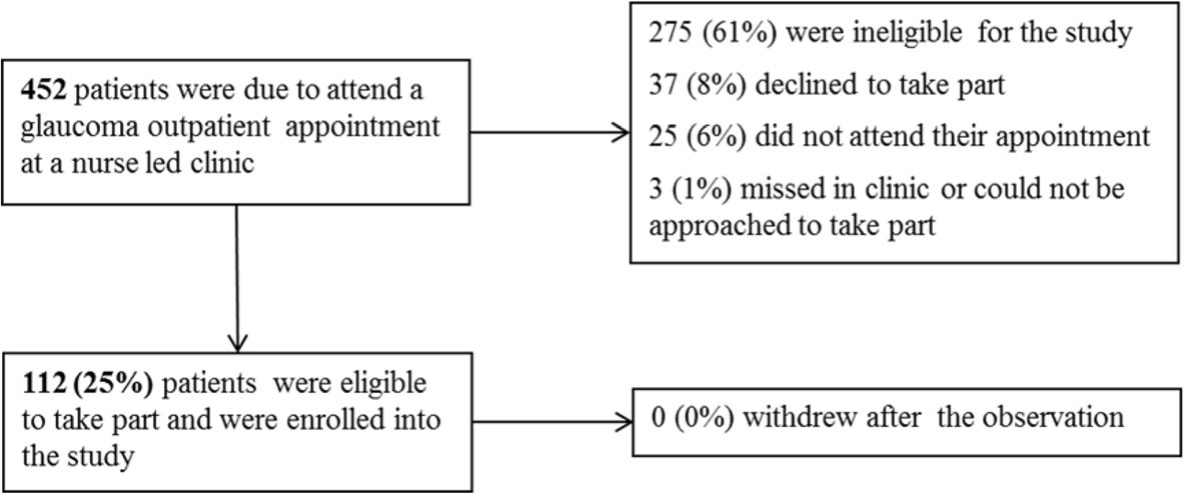
Patient recruitment flow for phase 1 of the study

All nurses from the study hospitals who provided nurse-led consultation consented to be observed (*n* = 9). A total of 112 patients consented to be observed at the three study sites with one hospital recruiting fewer patients than the other two because of staff sickness (hospital 1, *n* = 50; hospital 2, *n* = 43; hospital 3, *n* = 19). Figure 1 shows the patient recruitment pathway. The majority of the sample in phase 1 of the study were aged 61 or older, white, retired and had a diagnosis of COAG, and there were 10% more males in the study than females (Table 1). Eleven out of 112 patients had been treated for glaucoma for 6 months or less; none were new patients because these were seen by doctors.

**Table 1:** Demographic information on participants in phase 1 of the research

Demographic category	Response	112 patients
Mean age	–	71 years
Time since diagnosis	–	7.2 years
Gender	Male	55% (61)
	Female	45% (51)
Ethnicity	White	95% (106)
	Black	2% (2)
	Asian	4% (4)
Diagnosis	COAG	60% (67)
	OHT	21% (24)
	Suspected COAG	6% (7)
	NTG	9% (10)
	Not stated	4% (4)
Field loss	Yes	55% (61)
	No	38% (43)
	Don’t know	4% (4)
	Not stated	3% (3)
	Low test reliability	< 1% (1)
Employment status	Retired	75% (84)
	Employed full time	13% (14)
	Unemployed	3% (3)
	Employed part time	2% (2)
	Unable to work NOT due to an eye condition	3% (3)
	Not stated	4% (6)

*Key*: *COAG* chronic open-angle glaucoma, *OHT* ocular hypertension, *NTG* normal tension glaucoma

During clinic, the main areas that nurses discussed with patients were in relation to the management of the condition, while areas that were less likely to be mentioned to patients were the prognosis of the disease and information about support services (Table 2).

**Table 2:** Information provided to patients during phase 1 and phase 3

Category from the VIM tool	Phase 1	Phase 3
	112 patients	16 patients
Management—investigations, e.g. fields test	88% (99)	100% (16)
Self-management—importance of taking eyedrops	83% (93)	100% (16)
Management—regular monitoring	63% (70)	81% (13)
Management—appointment issues	63% (70)	94% (15)
Treatment—options for eyedrops	60% (67)	100% (16)
Risk—family	55% (62)	100% (16)
Diagnosis	54% (60)	100% (16)
Treatment—side effects	43% (48)	100% (16)
Prognosis—loss of sight	24% (27)	100% (16)
Driving	16% (18)	94% (15)
Support—help with eyedrops	13% (15)	100% (16)
Prognosis—symptomless	11% (12)	100% (16)
Prognosis—sight can’t be recovered	10% (11)	100% (16)
Support—support groups	10% (11)	94% (15)
Prognosis—when treated most don’t go blind	3% (3)	100% (16)
Registration of loss of sight	2% (2)	6% (1)

NB. Phase 3, registration of loss of sight, one patient was given information in response to an individual question which was not widely discussed with the whole group

*VIM* Provision of Vision Information Measurement tool

### Phase 2—training of nurses

A total of six nurses were invited to take part, one refused citing dislike of public speaking, and another was approached as replacement. Four of the nurses attended a 2-day workshop held at a local university and two received 1-day training provided at their hospital. All the nurses gave informed written consent.

### Phase 3—implementation and acceptability of the ‘Get a grip on your glaucoma’ course

#### Implementation

**Fig. 2:**
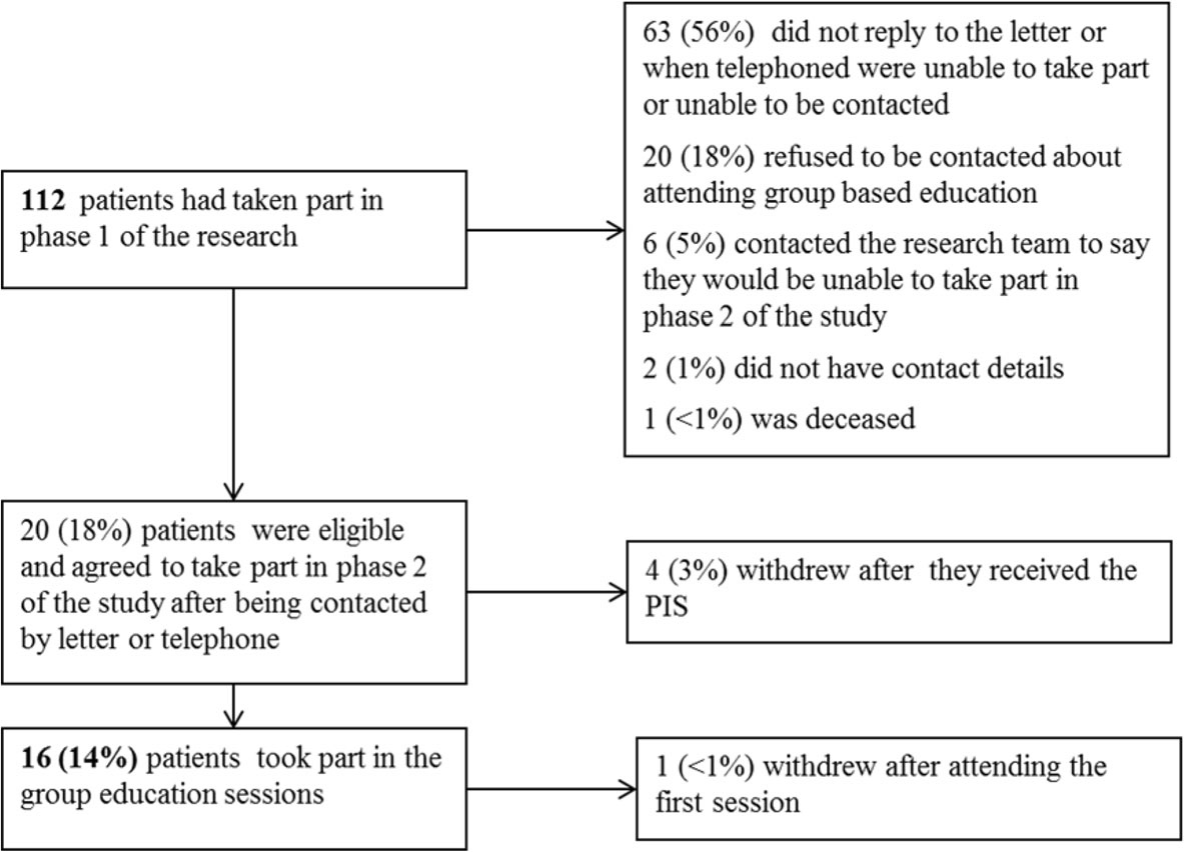
Patient recruitment flow for phase 3 of the research

Five nurses were observed delivering a total of three sets of two sessions of the ‘Get a grip on your glaucoma’ course (six sessions in total) to the patients in phase 3. Where possible, two nurses from the same hospital shared the delivery of the sessions together. One nurse who had attended phase 2 was unable to take part in phase 3 because of personal reasons. The other nurse from the same hospital delivered the two sessions on her own so in fact no patients needed to be rescheduled. Twenty patients were eligible and 16 attended (Fig. 2). There were variances to the planned delivery of the course at the hospital where the nurses received only 1-day training. This amounted to omissions of parts of the course in which the nurses had been trained to deliver. Reported reason for the omission was that they considered the patients’ would not benefit from them.

##### Outcomes

When the nurses delivered the group-based education, most items 14/16 on the provision of information clinical standard were delivered 100 or 94% of times to patients (Table 2).

#### Acceptability

Five out of 6 nurses and 6 out of 16 patients were also interviewed about the acceptability of the programme. One nurse was not interviewed as s/he did not deliver the programme, and patients declined to be interviewed because of lack of time. Four themes emerged from the interview data: Amount and quality of information, Opportunity to share experiences, Timing of the provision of information and A framework for teaching.

##### Amount and quality of information

Patients who were interviewed felt that the group sessions had given them more information than they had previously received during clinics:

….people of my generation and older I think they are reluctant to ask, especially when they see something medical almost on a pinnacle and “yes doctor”, they don’t feel able to ask and I do think that there have been things in these sessions that perhaps should be standard without asking. Patient 2, hospital 1.

Another patient explained because the clinics were so busy that the amount of information needed could not be routinely provided:

I appreciate when I come here it is busy. It is very, very busy. Patient 4, hospital 3.

Nurses also considered that they were not able to give the patients the amount of information that patients needed during routine clinic appointments.

….it has made us realise that we’ve got to give more information and, you know, when you’re working you, sort of, one part of you is watching the clock and, you know, and I think you’ve just got to give that bit more time to some people’ Nurse 2, hospital 3.

…You know, if there’s a problem, obviously, you have to take more time, I’m not saying, but even so when some patients come in and they know nothing and some are very informed, and it is difficult and there are things you want them to know and… Also, you can’t bombard them at a clinic visit with everything’ Nurse 2, hospital 1.

##### Opportunity to share experiences

One patient expressed how important it was to have the opportunity to talk to other people with the same condition as this does not happen normally in everyday life.

…yes, it’s nice to have other people with the same condition to talk to because I don’t think it’s very good on your own. You don’t meet people with the same condition otherwise or talk to people who have it, it’s very reassuring. Patient 9, hospital 1.

Similarly, another patient talked about the benefit of taking part in group-based education especially in terms of hearing each other’s experiences:

I think the group sessions were great because most of us were at different stages of the glaucoma… It was good to hear what other people felt or their problems or their issues… Patient 22, hospital 1.

##### Timing of the provision of information

They also considered that they would have liked to have received the information on glaucoma when they were first prescribed eyedrops as one patient said ‘I could have done with this months ago.’ Patient 4, hospital 2.

In a similar vein, nurses argued that:

…I do think if units can set up sessions like that and get patients very early on in the diagnosis you’re definitely going to improve compliance… Nurse 1, hospital 1

So there’s got to be a system put in place, I think, where they can…. just to come back to see the glaucoma nurses to go through everything… Nurse 1, hospital 2

##### A framework for teaching

The nurses discussed how the course provided them with a rationale and a framework for teaching patients:

…what it teaches you, as a practitioner, is to think that bit more about what you’re saying, you know and the information that you’re giving... Nurse 1, hospital 1

…I think we did it [provided education] before but we hadn’t got a structure and I think now we’ve got a structure haven’t we? Nurse 1, hospital 3

They reported that the course covered almost everything that patients ask about in clinic.

## Discussion

### What we found

This is the first study to consider how nurses might achieve a clinical standard for glaucoma information provision to patients. The interview data support the quantitative findings demonstrating the challenges involved in trying to impart sufficient information during routine consultations. These results suggest that patients received more information about their glaucoma in accordance with the clinical standard during the group-based education programme than they did during their usual clinical appointments. Specifically, our results show that implementing the ‘Get a grip on your glaucoma’ course will help nurses achieve the clinical standard for glaucoma information provision.

Nurses did not systematically assess the prior learning of patients, and since we did not collect data on length of consultation and time since diagnosis, we cannot say whether this was a factor in what nurses discussed with patients in their consultations. Neither can we say whether nurses tailored their consultation to what had been told patients in the past, but the results tell us that overall, little information was provided across the board because the clinics were too busy, patients do not like to ask and nurses have not previously been trained to deliver programmes.

Among other things, the study results show that this could be because the nurses did not have access to relevant educational resources or tools. If they could access the ‘Get a grip on your glaucoma’ course as a matter of routine then compliance with the guideline could improve. Other studies have noted that patient education tools are perceived as being necessary to the implementation of guidelines [15].

### Implications for a future trial

The results of this study demonstrate that the group-based education programme provides information above that normally provided in routine clinical appointments hence adding weight to its scope. Our findings show that nurses from three hospitals, once trained, can deliver group-based education which addresses the clinical standard which augers well for the implementation of a full randomised controlled trial. Our study also shows that nurses and patients report finding the course useful and thorough again adding evidence to the credibility of the trial intervention. However, our experience from carrying out the study has revealed some challenges which need to be borne in mind for a definitive trial.

### Strengthening the trial

Information gained from the observation phases and the preparations for the group-based education in each site has enabled the research team to construct a training package designed to facilitate these processes within a main study. The full 2-day training event will need to be utilised in a future trial so that there is equity and parity across all sites and to ensure rigour and fidelity to the intervention.

The response rate to the letter inviting patients to attend the group-based education programme in phase 3 was low. This was likely to be because there was a time lag of between of 3 months and 1 year between the offer of education and the delivery. This was caused by nursing staff sickness. A future trial design could complete all three phases at one hospital independently of the other hospitals. In the present study, we waited until phase 1 was finished across the three hospitals before moving onto phase 2. This was because we thought that to train the nurses together would be good for the exchange of information and experience; however, because of slow recruitment at one hospital in hindsight, it slowed down the start of the whole of phase 2. Thus, a future trial will need to ensure that there is minimal time between invitations to actual attendance on the course.

The participants in the present study were not new patients, but the interviews suggest that these too could benefit from the programme. If they had been included, perhaps the response rate may have been better [11]. Other research findings also report patients taking non-oral medication including eyedrops who are more recently diagnosed should be a target group for education programme [16]. It would appear that this group should be included in the protocol for a proposed trial.

As already stated, staff sickness contributed to the time lag between the invitation to attend the course and its actual delivery, and so when designing the trial, it will be essential to build in contingency plans to minimise the impact of sickness, for example to train more nurses than is actually required.

### Further adaptions to the clinical standard

The results show that patients often asked about other eye and systemic diseases or abnormalities and their relationship to glaucoma during routine glaucoma clinic appointments. For example, patients wanted to know whether there was a relationship between diabetes and glaucoma. The clinical standard should be amended to take these extra education needs into account by inserting under a new heading of ‘Other eye diseases – The relationship of glaucoma to other eye diseases or systemic conditions e.g. diabetes, cataract, dry eyes, and macular degeneration'.

## Conclusions

The group-based education programme, ‘Get a grip on your glaucoma’ course, provides patients with more information in line with a clinical standard than that routinely given in nurse-led clinics. Group-based education is acceptable to both patients and nurses and is viewed as a positive and appropriate means of giving patients the information they need to make informed decisions about their health.

In conclusion, valuable information was obtained about current information provision during routine nurse glaucoma clinics and the feasibility and acceptability of delivering patient information in line with a clinical standard by training nurses from multiple hospital sites. However, recommendations for a future trial would be to alter the trial design so that the research, training and delivery of group-based education are carried out independently between hospitals, that the training is consistently delivered to sufficient numbers of nurses and that there is minimal time lag between inviting patients and delivering the education, and new patients are included in the sample. With a few modifications, the evidence thus gathered lends support to the next step to design a definitive randomised controlled trial on the effectiveness of the intervention to improve adherence to glaucoma eyedrops.

## Appendix

**Table 3:** Provision of Vision Information Measurement tool (VIM)

Definition	Yes	No	N/A	Unable to answer
Diagnosis Specific condition				
Prognosis Its life-long implications for their prognosis for retention of sight				
Prognosis That COAG in the early stages and OHT and suspected COAG are symptomless				
Prognosis That most people treated for COAG will not go blind				
Prognosis That once lost, sight cannot be recovered				
Management The need for regular monitoring as specified by the healthcare professional				
Management Methods of investigation during assessment				
Management How long each appointment is likely to take and whether the person will need any help to attend (for example, driving soon after pupil dilation would be inadvisable)				
Risk factors For example, that glaucoma can run in families and that family members may wish to be tested for the disease				
Self-management The importance of the person’s role in their own treatment—for example, the ongoing regular application of eyedrops to preserve sight				
Treatment The different types of treatment options, including mode of action, frequency				
Treatment Severity of side effects, risks and benefits of treatment				
Support Support groups				
Support Information on how to access help with eyedrops and devices if required				
Driver and Vehicle Licensing Agency (DVLA) regulations				
Letter of Vision Impairment (LVI), Referral of Vision Impaired Patient (RVI) and Certificate of Vision Impairment (CVI) registration				
Other				
Other				
Other				
Other				
Other				

## Abbreviations

COAG: Chronic open-angle glaucoma
NICE: National Institute for Clinical Excellence
OHT: Ocular hypertension
VIM: Provision of Vision Information Measurement Tool
